# Percutaneous kyphoplasty assisted with/without mixed reality technology in treatment of OVCF with IVC: a prospective study

**DOI:** 10.1186/s13018-019-1303-x

**Published:** 2019-08-08

**Authors:** Peiran Wei, Qingqiang Yao, Yan Xu, Huikang Zhang, Yue Gu, Liming Wang

**Affiliations:** Department of Orthopaedics, Nanjing First Hospital, Nanjing Medical University, No. 68 of ChangLe Road, Nanjing, 210029 Jiangsu Province China

**Keywords:** Mixed Reality, PKP, IVC, OVCF

## Abstract

**Background:**

The purpose of this study was to assess the clinical outcome of percutaneous kyphoplasty (PKP) assisted with mixed reality (MR) technology in treatment of osteoporotic vertebral compression fracture (OVCF) with intravertebral vacuum cleft (IVC).

**Method:**

Forty cases of OVCF with IVC undergoing PKP were randomized into a MR technology-assisted group (group A) and a traditional C-arm fluoroscopy group (group B). Both groups were performed PKP and evaluated by VAS scores, ODI scores, radiological evidence of vertebral body height, and kyphotic angle (KA) at pre-operation and post-operation. The volume of injected cement, fluoroscopy times, and operation time were recorded. And cases of non-PMMA-endplates-contact(NPEC) in radiological evidence was also recorded postoperatively. The clinical outcomes and complications were evaluated afterwards. All patients received 10 to 14 months follow-up, with an average of 12 months.

**Result:**

This MR-assisted group (group A) acquired more about the amount of the polymethyl methacrylate (PMMA) injection and postoperative vertebral height and less about postoperative KA, fluoroscopy times, and operation time compared with the control group (group B) (*P* < 0.05). The VAS scores and ODI scores in both groups have improved, but more significantly in group A (*P* < 0.05). Also, more cases achieve both-endplates-touching of cement in group A (*P* < 0.05). And there are less of the loss of vertebral height, KA, and occurrence of re-collapse of the vertebra in group A during the follow-up (*P* < 0.05).

**Conclusion:**

PKP assisted with MR technology can accurately orientate the position of IVC area, which can be augmented by the balloon leading to more satisfied vertebral height improvement, cement diffusion, and pain relief.

**Trial registration:**

ClinicalTrials.gov Identifier: NCT03959059. Registered 25 September 2016.

## Key points

1. Destruction of membrane at the periphery of the IVC is probably the key point for distribution of cement to be interdigitated with the surrounding cancellous bone for the sake of better clinical efficacy.
2. The location of IVC in the vertebral body could be accurately acquired preoperatively from the CAD virtual anatomic images obtained by fusion of MRI and three-dimensional CT images, which was a great advantage for the guidance.
3. PKP assisted with MR technology could acquire real-time and accurate guidance to the IVC area during operation.

## Background

Percutaneous kyphoplasty (PKP) is an effective and currently widely used method for the treatment of osteoporotic vertebral compression fracture (OVCF), the procedure was done usually under local anesthesia, and the patient was well tolerated [1]. Early studies have shown that patients could obtain promising clinical outcome of immediate pain relief and functional improvement, especially among the elderly [2–6]. However, there is still a very number of patients who were unsatisfied with the result after operation. As for these patients, they complain about their unsatisfied or unchanged pain relief or even worse pain, which may indicate continued compression or a recurrent fracture in the treated vertebra. Previous studies have shown that intravertebral vacuum cleft (IVC) in acute OVCF was not a rare phenomenon [7–10] and it also has been considered as an important risk factor for persistent back pain and severe vertebral collapse and might be the main reason responsible for unsatisfied outcome after PKP [11, 12]. On magnetic resonance imaging (MRI), an IVC presents linear or cystic hypointensity similar to air on T1-weighted sequences and hyperintensity similar to cerebrospinal fluid on T2-weighted sequences [13, 14]. It also has been demonstrated [15, 16] that during the PKP procedure, instead of diffusing into the surrounding cancellous bone, the cement was formed into a solid lump that causes the fibrocartilaginous membrane at the periphery of IVC inhibited its dispersion. The fibrocartilaginous membrane can be broken by the balloon injected into the IVC area, leading to improvement of the cement diffusion [17, 18]. Therefore, in order to break the fibrocartilaginous membrane of IVC by the balloon during PKP procedure, so as to make the cement sufficiently diffuse into surrounding cancellous bone, we attempted to acquire accurate navigation to the IVC area.

Mixed reality (MR) is a combination of VR and AR in 3D applications [19], and it divorces digital reality and virtual digital images from the virtual world on the screen, so that three-dimensional virtual objects and the real world can be accurately combined [20, 21]. Previous studies have reported that MR technology has been used to show three-dimensional reconstructed model of all aspects, both preoperatively and intraoperatively; the muscles, blood vessels, and bones could be accurately seen; and the anatomical data could be measured [22]. Thus, it could help the surgeon to select the best operative program individually and precisely [21]. Under this technology, the real tissues and preoperative image data can be matched and fused to achieve real-time three-dimensional visual positioning and real-time display of the position of surgical tools, so as to carry out surgical navigation [23, 24]. The purpose of the present study was to evaluate PKP assisted with precise navigation by MR technology in the treatment of OVCF with IVC.

## Methods

### Patients

All patients participated in the trial and signed informed consent voluntarily. There was also no financial relationship between the investigators and study participants. Patients were required to meet the following inclusion criteria: (1) single-level OVCF in the thoracic and lumbar levels (T10-L4) [25], suffering with severe back pain, and (2) OVCF without damaged vertebral posterior wall and nerve lesion; (3) the patient age was more than 50 years old, and diagnosis of osteopenia or osteoporosis was confirmed by bone densitometry; and (4) all patients underwent plain radiography, computed tomography (CT), and MRI before surgery, with IVC presented in MRI. From June 2017 to September 2017, there were 40 patients who met the criteria above; who participated the trial, aging from 50 to 95 years old; and were randomized into MR technology-assisted group (group A) and traditional C-arm fluoroscopy group (group B), 20 cases for each group.

Approval was obtained from the Institutional Review Board of Nanjing First Hospital prior to performing the study.

### Assessment of clinical parameters

Several parameters including age, sex, and bone mineral density (BMD) of the two groups have been evaluated preoperatively. Operation duration, fluoroscopy times, and volume of cement injected to both groups were recorded respectively. Symptoms of two groups were evaluated by visual analog scale (VAS) score of back pain and Oswestry Disability Index (ODI) of movement function. Immediate postoperative, 1-, 3-, 6 months, and 1 year VAS and ODI scores were compared.

### Assessment of radiological parameters

**Fig. 1:**
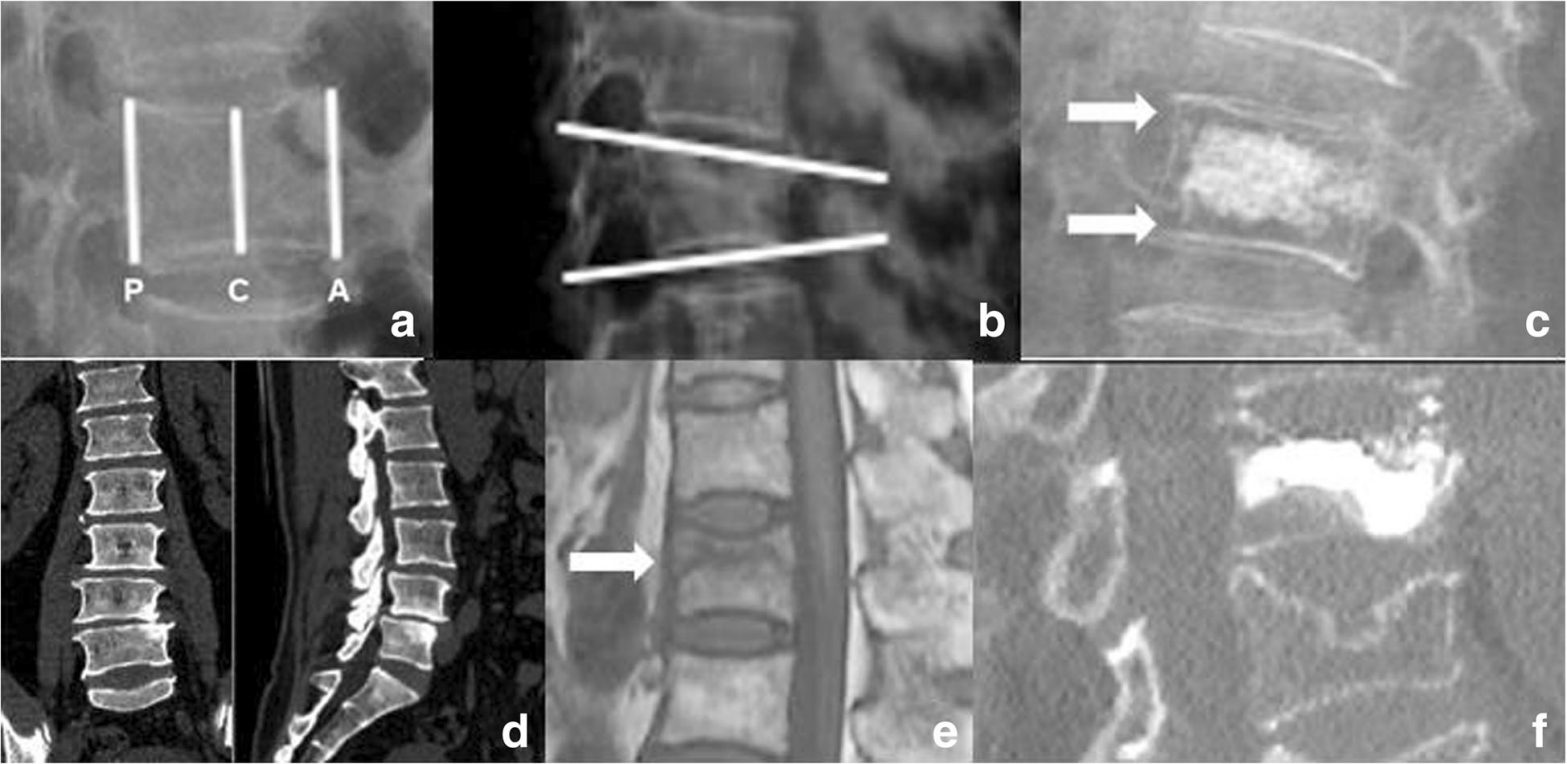
**a** (A): anterior vertebral height, (C): central vertebral height, (P): posterior vertebral height. **b** Kyphotic angle (KA). **c** Cement non-contact both endplates (NPEC). **d** CT images. **e** MRI image of OVCF with IVC. **f** Postoperative CT images, some part of the vertebra is not filled with cement

**Fig. 2:**
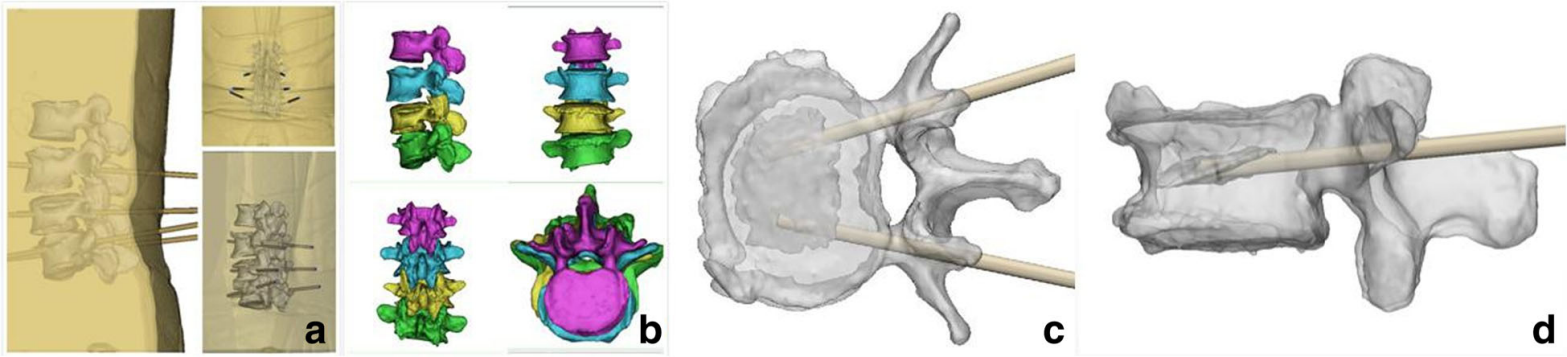
**a**–**d** CAD virtual anatomic images and the position of IVC in the merged images of reconstructed vertebra

We measured preoperative and postoperative (1 day, 1-, 3-, 6 months, and 1 year) radiological parameters including kyphotic angle (KA) from sagittal radiographs, and anterior, central, and posterior vertebral height; anterior/posterior height ratio was defined as relative anterior vertebral height, and central/posterior height ratio was defined as relative central vertebral height (Fig. 1a). Kyphotic angle (KA) was formed by the upper and lower endplates of the fractured vertebral body (Fig. 1b). Patients underwent CT scanning pre- and postoperatively (Fig. 1d, f). IVC was presented in MRI image (Fig. 1e). And cases of cement touching both endplates in each of the two groups were recorded postoperatively (Fig. 1c). In group A, patients also required accurate three-dimensional images to confirm the location of IVC in the vertebral body, which was obtained by computer-aided design (CAD) of preoperative MRI and three-dimensional CT images, for the purpose of navigation by MR technology (Fig. 2).

### Surgery

All of the surgeries were performed by one group of surgeons and adopted bipedicular approach in a prone posture under local anesthesia (1% lidocaine) in all cases.

**Fig. 3:**
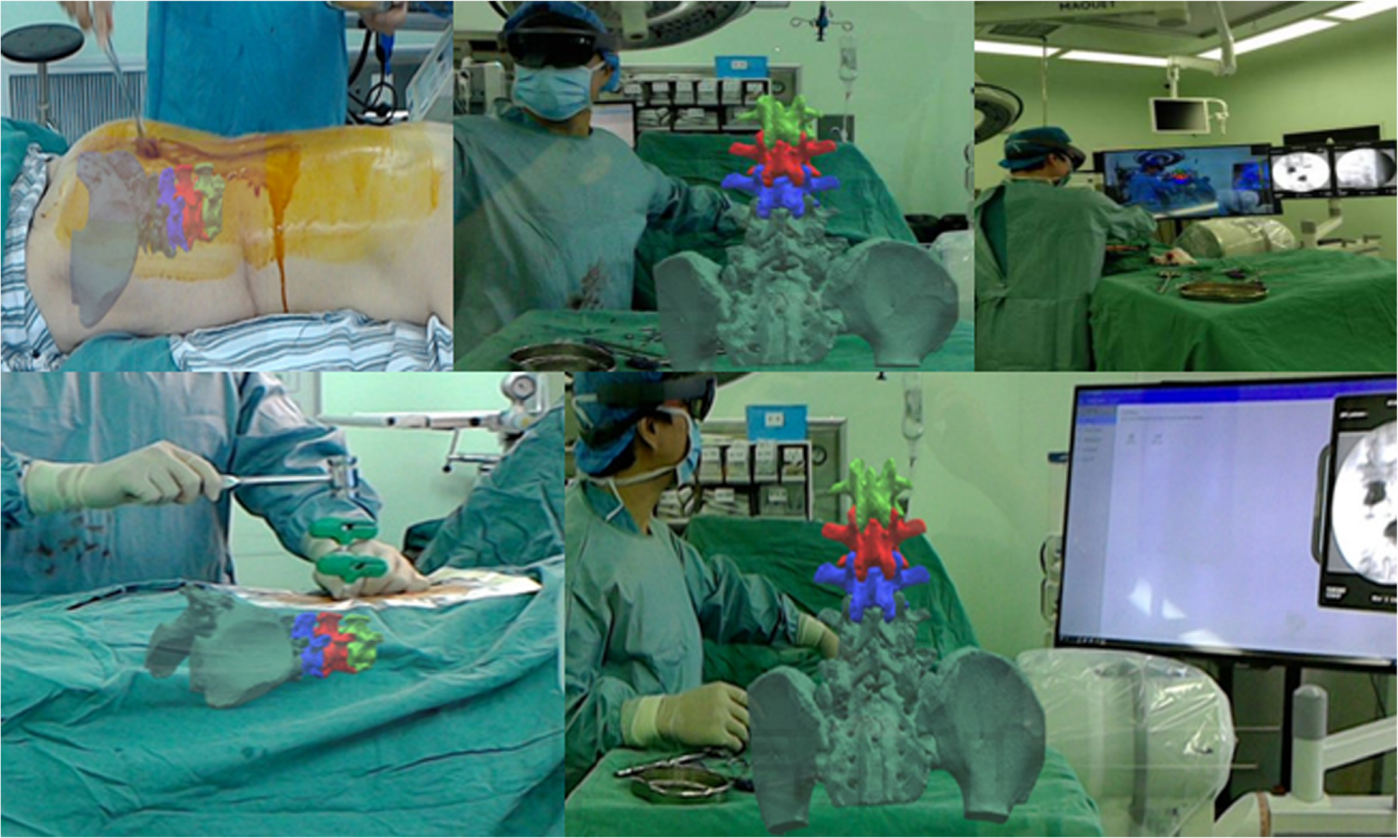
Procedures of PKP with MR technology and CAD virtual anatomic images

Group A: All the PKP operations were performed by one group of surgeons. Persons were in the prone position with local anesthesia (1% lidocaine), through bilateral transpedicle puncture approach. Cases in MR-PKP group (group A) underwent dual-source 64-slice CT volume scanning (Siemens Sensation, Germany) with a slice thickness of 1 mm and a scanning matrix of 512 × 512. Input the data obtained into the M3D digital medical software (Bahulu, Shanghai Front Computing Company, China); hence, the three-dimensional model was obtained by using its threshold segmentation and regional growth functions. The responsible vertebral body and adjacent vertebral body were labeled with different colors, the location and direction of pedicle insertion were pre-designed, and the three-dimensional virtual digital CAD image was constructed (Fig. 2). Before the operation, the CAD image was imported into the computer of MR system (Medivi, Changzhou, China). Doctors wore AR glasses HoloLens (Microsoft, USA). As the original data for CT reconstruction was taken in supine position, the patient is placed in prone position during operation. We need C-arm fluoroscopy during operation to adjust the CAD image to optimally match the patient in prone position. X-ray fluoroscopy was performed, and the spatial registration of virtual image and patient’s body was completed by manual positioning. With MR technology and C-arm fluoroscopy, these 3D virtual images can be accurately combined with the patient, so the surgeon could see the spinal 3D virtual images (CAD images) accurately in the patient’s body. Through this combination of virtual images and real body, also combined with C-arm fluoroscopy, needles were accurately inserted through bilateral pedicle of the injured vertebra until the tip was optimally positioned in the IVC. Then the needles were removed from the trochar, and a balloon was placed into the channel and inflated in order to augment the IVC area, elevate the endplate, and restore the height of the vertebral body. Cement (poly methyl methacrylate, PMMA) was injected into the IVC area for attempting to make the cement to be sufficiently interdigitated with the surrounding cancellous bone and complete filling of the cleft, maximizing the stabilization of the IVC and fracture (Fig. 3).

Group B: Insertion of the transpedicular access was performed bilaterally under C-arm fluoroscopic guidance. Combined with the X-ray, CT, MRI images preoperatively and the C-arm fluoroscopic images, the tip of needle was set to the most possible area of the IVC, the needle was removed and a balloon was inserted to the position, which was inflated and expanded to restore the height of the vertebra, and then the cement (PMMA) was injected. The filling and diffusion of bone cement were observed while injection was carried out. And the injection was stopped when the bone cement reached the posterior edge of the vertebral body.

### Statistical analysis

SPSS V.11.0 statistical software was used for analysis. Comparisons were made before and at each postoperative follow-up appointment. Qualitative characteristics of groups were assessed using one-way or repeated measures ANOVA and *t* test. *χ*^2^ tests were performed for categorical variables. *P* < 0.05 was considered statistically significant.

## Results

Forty patients (M/F 13:27) were randomly categorized into two groups, each group with 20 cases. The mean age of the patients was 73.48 ± 8.72 years in group A and 74.02 ± 8.85 years in group B, and the length of follow-up ranged from 10 to 14 months (median 12 months). There was no significant difference between the two groups in baseline parameters including age, sex, BMD, VAS, ODI, vertebral height, KA, level of injured vertebra, and the time from injury to surgery (*P* > 0.05, Table 1).

**Table 1:** Basic characteristics and preoperative data of the two groups

Patient characteristics	Group A	Group B	*P*
Age (years)	73.48 ± 8.72	74.02 ± 8.85	0.97
Women, *n* (%)	14 (70%)	13 (65%)	0.77
BMD score	− 4.35 ± 0.94	− 4.44 ± 0.76	0.89
VAS scores	8.38 ± 1.28	8.14 ± 1.35	0.31
ODI scores	83.95 ± 8.96	84.27 ± 7.48	0.13
Relative anterior vertebral height (%)	77.51 ± 5.91	75.72 ± 6.73	0.79
Relative central vertebral height (%)	56.74 ± 4.13	58.28 ± 3.62	0.96
KA (°)	14.02 ± 4.06	12.78 ± 4.75	0.39
Time from injury to surgery (number of patients)			
1 day	0	1	0.97
2 days	0	1	
3 days	15	14	
4 days	2	2	
5 days	3	2	
Level of injured vertebra (number of patients)			
T10	1	1	0.66
T11	1	1	
T12	5	6	
L1	6	7	
L2	4	3	
L3	2	2	
L4	1	0	

The operation duration and fluoroscopy times in group A were less than those in group B (*P* < 0.05). The amount of PMMA injection during MR guidance (group A) was more than that during C-arm fluoroscopic guidance (Table 2, *P* < 0.05).

**Table 2:** Postoperative data of the two groups

	Group A	Group B	*P*
Operative time (min)	25.12 ± 5.36	45.14 ± 3.86	< 0.05
Fluoroscopy times	31.87 ± 9.77	76.94 ± 8.65	< 0.05
Amount of cement injection (ml)	6.41 ± 0.54	4.22 ± 0.35	< 0.05
Cases of cement of both-endplates-contact	13	6	< 0.05
Relative anterior vertebral height (%)	92.42 ± 5.14	71.21 ± 4.83	< 0.05
Relative central vertebral height (%)	79.74 ± 3.92	60.53 ± 3.31	< 0.05
Improvement of relative anterior vertebral height (%)	12.81 ± 4.76	6.94 ± 3.42	< 0.05
Improvement of relative central vertebral height (%)	19.71 ± 2.82	3.42 ± 3.57	< 0.05
KA (°)	7.11 ± 3.01	10.89 ± 2.91	< 0.05
Change of KA (°)	7.11 ± 1.21	3.72 ± 1.53	< 0.05

The postoperative VAS and ODI scores were followed about 1 year. Immediately after surgery, the mean VAS and ODI scores of all patients in both of the two groups decreased significantly (*P* < 0.05), but the scores decreased more in group A (*P* < 0.05). However, we observed a gradual significant increase in VAS and ODI scores during the 1-year follow-up after surgery in group B. The VAS and ODI scores were significantly higher in group B than those in group A at the 1-year postoperative follow-up (*P* < 0.05). The VAS and ODI scores in group B immediately after surgery were 2.58 ± 1.09 and 29.11 ± 8.07, respectively, and increased to 3.42 ± 1.16 and 35.07 ± 9.1 at 1 year, respectively (Tables 2 and 3).

**Table 3:** VAS and ODI scores and imaging parameters during 1 year follow-up

	Group A	Group B
VAS		
Immediate postoperative	1.37 ± 1.01	2.58 ± 1.09
1 month	1.33 ± 1.03	2.96 ± 1.15
3 month	1.39 ± 1.04	3.17 ± 1.20
6 month	1.36 ± 1.08	3.21 ± 1.08
12 month	1.31 ± 1.12	3.42 ± 1.16
ODI		
Immediate postoperative	18.97 ± 7.79	29.11 ± 8.07
1 month	19.05 ± 8.03	31.34 ± 8.73
3 month	19.23 ± 8.77	33.68 ± 7.85
6 month	19.51 ± 8.02	33.94 ± 8.67
12 month	19.87 ± 8.34	35.07 ± 9.10
Relative anterior vertebral height (%)		
Immediate postoperative	92.42 ± 5.14	71.21 ± 4.83
1 month	92.21 ± 4.49	68.88 ± 5.01
3 month	91.75 ± 6.44	66.35 ± 5.94
6 month	91.69 ± 5.02	63.81 ± 6.23
12 month	91.58 ± 5.34	61.72 ± 6.96
Relative central vertebral height (%)		
Immediate postoperative	79.74 ± 3.92	60.53 ± 3.31
1 month	79.19 ± 3.88	58.91 ± 3.56
3 month	78.77 ± 4.07	58.21 ± 3.87
6 month	78.65 ± 3.98	57.37 ± 3.61
12 month	78.31 ± 4.11	56.96 ± 3.71
KA (°)		
Immediate postoperative	7.11 ± 3.01	10.89 ± 2.91
1 month	7.73 ± 3.11	11.56 ± 3.34
3 month	7.78 ± 2.89	11.88 ± 2.85
6 month	7.85 ± 3.08	11.96 ± 3.41
12 month	8.01 ± 3.67	12.47 ± 3.28

Vertebral height was defined as relative anterior vertebral height (anterior/posterior height ratio) and relative central vertebral height (central/posterior height ratio). KA and preoperative reduction in height of the vertebra were not significantly different in the two groups (*P* > 0.05). However, postoperative height of the vertebra (relative anterior height, relative central height) was significantly taller in group A than in groups B (Table 2, *P* < 0.05). Vertebral kyphotic angle (KA) in group A was also significantly lower than that in group B (Table 2, *P* < 0.05). Cases of cement-both-endplates-contact were 13 in group A and 5 in group B, which was significantly different (*P* < 0.05) (Tables 2 and 3).

### Complications

No cement leakage, neurologic deficits, re-fracture of the treated vertebra, and other complications were observed in these two groups. There was one case of adjacent vertebral fracture in group B, while none in group A. The perioperative mortality was 0%.

## Discussion

IVC in the fractured vertebra has been reported to be the main reason to intractable pain of OVCF and unsatisfied pain relief after PKP [26]. And IVC may cause pseudarthrosis of the vertebra leading to spinal instability, which may give rise to the severe chronic pain. Therefore, it was thought to be a good indication for PKP to acquire pain relief and spinal stabilization through filling IVC with cement. However, Heo et al. [15] found that the cement cannot be diffused to be interdigitated with the surrounding cancellous bone because of the inhibition by the fibrocartilaginous membrane of IVC. It formed into a solid lump which may induce greater stress to the surrounding cancellous bone, leading to recollapse or refracture [15, 18, 27]. It has been reported that distribution of the cement is more important than the volume injected, specifically acquiring the cement touching both endplates may be more crucial for stabilization of the compression fracture [28–31]. Both the superior and inferior endplates should be touched with cement to provide complete support of the vertebra to prevent continuing collapse and recurrent fractures [9, 11, 32–34]. Biomechanical studies demonstrated that better diffusion of cement distributes the load of the fractured vertebrae [35, 36]. Breaking the peripheral membrane, filling the cleft, and making the cement be interdigitated with the surrounding cancellous bone are the key point to pain relief and preventing further collapse [37]. However, the needle tip in previous study was still traditionally placed in the IVC area and lacks accurate navigation and guidance.

Therefore, we combined MR technology with the real patient in order to achieve accurate navigation to IVC area of OVCF. Mixed reality (MR) is a technology that divorces digital reality and virtual digital images from the virtual world on the screen, so that three-dimensional virtual objects and the real world can be accurately combined, which enables accurate replication of patient anatomy. Using the presented methodology, patient anatomy and surgical instruments can be aligned correctly in spatial relation to each other [38, 39]. Combining 3D reconstruction and MR technology is a new mode of production, which is applied in many fields because of the advantages of visualization, plasticity, and rapid printing [40–42]. Moreover, it can also help surgeons to achieve accurate navigation and position in spinal surgery [43, 44].

In our study, the location of IVC in the vertebral body could be accurately acquired in CAD anatomic images preoperatively as a result of fusion of MRI and three-dimensional CT images, and the CAD virtual digital images could be accurately combined with the patient’s body during operation, which was a great advantage for the guidance. Combined with C-arm fluoroscopic images during surgery, MR technology could assist surgeons to acquire real-time and accurate guidance to the IVC area, reducing operation time and numbers of C-arm fluoroscopy and increasing the accuracy and efficacy of surgery.

Our study also demonstrates that mixed reality (MR) technology can change the bottleneck of unarmed puncture in IVC-PKP surgery. The vertebra can be reconstructed and restored by three-dimensional CT reconstruction before operation, so that the location of IVC lesions can be displayed more intuitively in front of doctors. During the operation, the surgeons wore HoloLens glasses and obtained holographic image information, so that the patient’s virtual 3D digital model overlapped completely with the patient’s lesion site; thus, the doctor has a new way of operation [45].

With the assistance of MR in our study, the accuracy of puncture was significantly higher than that of traditional methods during the PKP procedure, especially in some patients with lesions of the vertebrae. The vertebral body may be accompanied by mild lateral bending, rotation, the formation of a bridge, and so on, which increase the difficulty of the operation, and the entry point and direction are difficult to control, especially in patients with IVC. Combined with MR, it can assist virtual positioning, give doctors the correct positioning point and direction when punctured, make the procedure more accurate and safer, is easy to operate, and obviously reduce the number of fluoroscopy and operation time. Elmi-terander et al. [46] confirmed that combined with intraoperative 3D image, navigation technology is expected to replace intraoperative X-ray or fluoroscopy in the process of thoracolumbar pedicle puncture and has high accuracy and safety.

Our study also indicates that the number of X-ray irradiation of C-arm machine in MR-PKP group was significantly lower than that in the traditional group. In the past, C-arm machine was needed to judge the position of the needle many times during operation, which was harmful to doctors. However, with MR navigation technology, the number of C-arm machine used in operation could be significantly reduced, and the injury to doctors and patients could also be obviously reduced.

In our study, we also found that the amount of cement injected and relative vertebral height in the MR-assisted group increased more compared with the control group. The presence of the peripheral membrane may prevent the diffusion of cement; thus, some part of the vertebra cannot be filled with cement [47], leading to the smaller volume injected in group B (Fig. 1f). The vertebral height was reconstructed by balloon and prone postural reduction, without sufficient cement reinforcing, especially touching both endplates; the vertebral height in group B may not be maintained and might be lost after operation [47–49]. Daisuke et al. [50] have reported that increased cement injection may be due to the increased balloon expansions, and this increased amount of cement injection may affect postoperative vertebral height improvement.

However, delayed reaction, small field of vision, dim brightness, and insufficient stability of binocular shared vision of MR technology inevitably lead to the need of C-arm fluoroscopy assistance. In the specialized soft- and hardware, the cost was relatively higher.

## Conclusion

In conclusion, MR technology was adopted as an efficient method for navigation and position. Compared with the traditional procedure of PKP, it acquired an accurate position of IVC in OVCF and preferable clinical outcomes. We consider this technology as a perfect approach for guidance in minimally invasive surgery.

### Limitations

1. This research lacks of biomechanical study, and the sample size is too small; this also needs long-term follow-up and research.
2. Although with fused CAD anatomic images preoperatively, MR technology cannot acquire real-time MRI images of IVC during operation.
3. For the disadvantages of delayed reaction, small field of vision, dim brightness, and insufficient stability of binocular shared vision, MR technology still needs C-arm fluoroscopy assistance.

## Data Availability

The datasets used and/or analyzed during the current study are available from the corresponding author on reasonable request.

## Abbreviations

BMD: Bone mineral density
CAD: Computer aided design
CT: Computed tomography
IVC: Intravertebral vacuum cleft
KA: Kyphotic angle
MR: Mixed reality
MRI: Magnetic resonance imaging
ODI: Oswestry Disability Index
OVCF: Osteoporotic vertebral compression fracture
PKP: Percutaneous kyphoplasty
PMMA: Poly methyl methacrylate
VAS: Visual analog scale
